# A Longitudinal Study on the Effects of Parasocial Relationships and Breakups With Characters of a Health-Related TV Show on Self-Efficacy and Exercise Behavior: The Case of The Biggest Loser

**DOI:** 10.1177/21674795211045039

**Published:** 2021-09-20

**Authors:** Perina Siegenthaler, Tanja Aegerter, Andreas Fahr

**Affiliations:** 1 Department of Communication and Media Research, 27211 University of Fribourg, Fribourg, Switzerland

**Keywords:** parasocial relationship, parasocial breakup, self-efficacy, exercise behavior, entertainment-education, longitudinal study

## Abstract

Overweight is one of the major health-related challenges in industrialized countries and mostly preventable through a healthy diet and regular engagement in physical activity. Health communication practitioners and researchers, therefore, started using the media’s persuasive potential by creating entertainment-education (E-E) programs that promote healthy nutrition and exercise. By observing the characters in E-E programs, audience members can learn vicariously and eventually develop personal bonds with them. The current study investigates the effects of parasocial relationships (PSRs) with characters of a health-related E-E show, as well as the impact of parasocial breakups (PSBUs) on health-relevant outcomes. Using the setting of the show The Biggest Loser (TBL), we conducted a quasi-experimental longitudinal field study. Participants (*N* = 149) watched shortened episodes of the show once a week for 5 weeks. Results showed that PSRs with the reality TV characters did not increase over time and after repeated exposure. Findings furthermore suggest that PSR did not influence self-efficacy perceptions or exercise behavior over time. Parasocial breakup distress intensity was neither related to self-efficacy nor to exercise behavior. Interpretations of these findings and implications for better understanding the effects of PSRs and PSBUs are discussed.

Fighting overweight and obesity is one of the major health-related challenges faced by societies in industrialized countries. Increased weight negatively influences the perceived quality of life (Kroes et al., 2016) and increases health problems (World Health Organization, 2020). In light of these developments and their severe impacts, overweight and obesity are important topics to address in health communication, especially as they are mostly preventable through a healthy diet and regular engagement in physical activity (World Health Organization, 2020). Although people are aware of the relevance of constant exercise, they lack the motivation to perform physical activity on a regular basis (Orji, 2014; Oyibo et al., 2018). Therefore, health communication practitioners and researchers started using the persuasive potential of the media and, in particular, television, by creating entertainment-education (E-E) programs (Moyer-Gusé, 2008; Singhal & Rogers, 1999) that promote healthy nutrition and exercise (Nabi & Thomas, 2013; Y. Tian & Yoo, 2015).

One assumption underlying the positive effects of E-E programs is that people learn vicariously by observing models (Bandura, 1986, 2001) such as media characters. Over time and after repeated exposure, viewers might develop so-called parasocial relationships (PSRs) with those characters (R. B. Rubin & McHugh, 1987). These one-sided imaginary bonds are supposed to enhance attention to the information provided in the E-E message (Klimmt et al., 2006). Eventually, the desired persuasive effects of the message are increased (e.g., Hoffner & Cohen, 2012). Due to the typical course of E-E TV programming, shows go off the air, and media characters are exchanged. Parasocial relationships with the media characters can, thus, come to an end which is termed as parasocial breakup (PSBU). If PSRs are meaningful and have effects on health-relevant outcomes, it can be argued that also the ending of such relationships has some impact on health-relevant outcomes. For example, previous research showed that PSBUs can lead to emotional distress (J. Cohen, 2004; Eyal & Cohen, 2006); however, little is known about the effects of these PSBUs on health-relevant behaviors in particular. Therefore, the objective of the present study was to investigate how PSRs, as well as breakups with media characters, affect health-relevant outcomes. Studies assessing PSRs as the underlying mechanism explaining post-exposure effects have relied heavily on data obtained from cross-sectional surveys. In order to close this research gap, a longitudinal design was executed using the health-related reality TV show The Biggest Loser (TBL) as a setting to analyze the development and effects of PSRs and breakups on efficacy perceptions and exercise behavior. We chose TBL because this reality TV show addresses the issues of overweight and obesity by promoting weight loss through physical exercise and a restrictive diet in an entertaining media format displaying ordinary people with whom the audience members can develop personal bonds.

## Physical Exercise and Health Communication

According to the World Health Organization (WHO), overweight and obesity rates of women and men over 18 years old rise constantly. Causes for this development are lifestyles lacking exercise and physical activity, as well as unhealthy diets (World Health Organization, 2020). Increased weight has severe consequences on personal health. For example, obesity raises the risk of metabolic diseases like Diabetes Type II or cardiovascular disorders (World Health Organization, 2020). Furthermore, on an aggregated level, obesity negatively affects the economic performance of a country and substantially increases social costs (Dobbs et al., 2014; Lehnert et al., 2013).

Physical activity is proven to be a fundamental means to avoid or reduce overweight and obesity as well as its negative consequences. Regular exercise increases total energy expenditure, and a negative energy balance is promoted when caloric intake remains lower than energy expenditure (Swift et al., 2018). Thus, physical activity can reduce overweight and obesity levels. However, surveys show that one in four adults worldwide does not even meet the global recommendations for physical activity of 150 minutes of moderate-intensity activity per week (World Health Organization, 2019). Moreover, previous research found that most individuals recognize the relevance and benefits of exercising regularly. However, they lack the motivation to put these behaviors into practice (Orji, 2014; Oyibo et al., 2018; for an overview, see, Rhodes & de Bruijn, 2013) and maintain regular exercise regimens (Zhou & Krishnan, 2019). Therefore, the challenge of how to increase individuals’ willpower to start or maintain regular physical activity is of significant interest in the area of persuasive health communication and has received attention since the 1970s (Bauman et al., 2006). Such persuasive messages may motivate recipients to reject the advice given in a health-related message because they feel as if their autonomy is threatened. In order to reduce the risk of adverse effects, health communication can be designed in a way that health and educational information is embedded in story plots, with implicit intent to persuade (L. Shen et al., 2017). Media producers and researchers, thus, develop E-E messages that promote healthy behaviors (Tukachinsky & Tokunaga, 2013) in order to influence and reinforce various health outcomes (Moyer-Gusé, 2008).

## Entertainment-Education and Parasocial Relationships

Entertainment-education programs aim at generating socially desirable effects among the audience (Papa et al., 2000) by placing educational content in entertainment messages (Singhal & Rogers, 2002). In health communication contexts, these formats embed health issues into programs to influence audiences’ knowledge, beliefs, attitudes, or behaviors with respect to the specific health-related topic (Nabi & Thomas, 2013). The persuasive intent is inherent in these programs (Behm-Morawitz et al., 2017), but storylines are added for their “dramatic appeal” (Moyer-Gusé, 2008, p. 409) in order to become attractive to the target audience. In this way, E-E programs can reach a broad audience, particularly people who do not actively seek health information themselves (Fromm et al., 2011).

One assumption underlying the positive effects of E-E is that the audience learns information about healthy practices through the media characters, which should eventually lead to improved behavior (Nabi & Thomas, 2013). This reasoning is rooted in Social Cognitive Theory (SCT), which assumes that individuals are more likely to perform a behavior they have seen demonstrated than one that was only recommended. Thus, by observing a model, individuals adopt new attitudes, beliefs, or behaviors (Bandura, 1986; 2001; Keller et al., 1999). For instance, research showed that the influence of models is a crucial factor in starting and keeping up physical activities (e.g., G. L. Smith et al., 2017; Unger & Johnson, 1995; Zhang et al., 2015). Thus, health behavior change can be initiated by vicarious experience, that is to say, by observing a person who successfully performs the respective behavior (Luszczynska & Schwarzer, 2005).

Studies on modeling identified several characteristics that can influence the audience member’s motivation, for example, perceived similarity to the model (Bandura & Walters, 1963; Rosekrans, 1967; Schunk & Di Benedetto, 2020). Therefore, successful E-E relies on media characters with whom the audience members can develop personal emotional and cognitive bonds (Moyer-Gusé, 2008), so-called PSRs (Horton & Wohl, 1956). Parasocial relationships are experienced subjectively by the viewer and, thus, remain one-sided and imagined (Schramm & Hartmann, 2008; Slater et al., 2017). Nevertheless, PSRs show similarities to real relationships with regard to sociability, sympathy, and intimacy (Gleich, 1997). It has been shown that PSRs can be more important than acquaintances but less important than good friends (Koenig & Lessan, 1985). The authors conclude that “quasi-friend” is an appropriate term to describe the relationship between audience members and a parasocial partner. Research also found that relationships with media characters have the same relationship qualities as a relationship with a good neighbor (Gleich, 1996).

In particular, reality TV shows allow viewers to form such connections because they cast individuals who appear to be ordinary people to whom audience members can easily relate (Nabi et al., 2003). Furthermore, as reality programs are less scripted, audience members have the feeling of watching real people presenting themselves and showing their true character (Hall, 2009). In addition, the displayed characters often express (presumed) spontaneously and unscripted emotions directly to the camera, which further fosters the connection between the media character and the viewer (Ebersole & Woods, 2007). Looking at the audience members directly, in turn, fosters parasocial interactions (PSIs) (Hartmann & Goldhoorn, 2011), which may initiate a loose parasocial relationship. This relationship may motivate the recipients to seek repeated contact with the character (Hartmann, 2017). During repeated exposure, PSRs with reality TV show characters should increase and the characters should become incorporated into the social networks of the audience members (Giles, 2002). As such, we hypothesize that PSRs with the media characters will grow over time (H1).

To date, many studies in health communication have focused on the influence of PSI on health-related outcomes such as behavioral intention (Jeong & Park, 2015) or behavior (Brown & de Matviuk, 2010). Parasocial interaction describes an imaginary interaction between the audience member and the media character that is characterized by feelings of reciprocity and takes place during media exposure (Schramm & Hartmann, 2008). Parasocial relationships, in contrast, also occur beyond the exposure situation. This means the audience member feels a connection with the media character, even when they are not consuming media displaying the respective character (Tukachinsky & Sangalang, 2016). In contrast to PSI (e.g., Kosenko et al., 2016; Y. Tian & Yoo, 2015), there is much less information about the effects of PSRs in health communication. To our knowledge, there are no studies analyzing the development of PSRs with media characters from health-related media formats over time and testing the effects of these connections outside the exposure situation. Hence, in the present study, we are especially interested in the effects outside the context of a particular media exposure instance and the evolution of PSRs over time.

## Effects of Parasocial Relationships on Self-Efficacy and Behavior

Seeing others successfully perform a behavior can increase beliefs in one’s own ability to successfully perform that behavior (i.e., self-efficacy; Luszczynska & Schwarzer, 2005; Resnick et al., 2002). In turn, the stronger the self-efficacy perceptions are, the more likely individuals will initiate and maintain a given practice (de Graaf & van Leeuwen, 2017). Indeed, perceived self-efficacy has been found to be an important factor in forming the intention to exercise and persisting with physical activity (McAuley, 1993; Rodgers et al., 2002; Rovniak et al., 2002).

Currently, there are no data on the effects of PSRs on self-efficacy. However, studies in the area of PSI show a positive relationship with self-efficacy. For example, (Y. Tian & Yoo, 2015) found that PSI is positively associated with exercise self-efficacy. The authors assume that recipients with more PSI observe the media characters from a third-person perspective and see them as “friends” who are trying to lose weight. The audience members would tend to explain the media characters’ behaviors as a function of the actors’ true dispositions. In turn, they reflect their own characters and are more likely to feel able to perform physical activities, as their quasi-friends can.

Transferred to PSRs, when the media character is perceived as a friend, audience members learn from observing this role model. This form of vicarious learning results in increased perceived self-efficacy. Specifically, audience members who connect strongly with the character are expected to be highly motivated to engage in exercise behavior and increase their own ability to perform that behavior successfully. Therefore, we assume that individuals who develop stronger PSRs with reality TV show characters will report more increase in self-efficacy perceptions over time than individuals who develop weaker PSRs (H2a).

Health behavior change might be triggered by vicarious experience if a (media) character models a desirable health behavior (Luszczynska & Schwarzer, 2005) and is rewarded for that. The show TBL offers audience members possibilities to observe the positive consequences of exercising, such as losing weight, feeling good, and gaining recognition from physical activity. Therefore, recipients learn that this behavior is likely to generate desired outcomes and are motivated to perform this behavior as well. Mediated observation of exercise behavior can thus influence behavior as if it was a real-life observation (de Graaf & van Leeuwen, 2017).

As with self-efficacy and PSR, research to date has not yet systematically determined the effects of PSR on exercise behavior. Regarding the effects of other health behaviors, Hoffner and Cohen (2012) found that stronger PSRs with a media character with mental illness enhanced willingness to seek treatment for people with obsessive-compulsive disorders. Previous research in the area of PSI found that PSIs with media characters increased athletic and healthy behavior (e.g., Sakib et al., 2020; Y. Tian & Yoo, 2015). Additionally, a recent meta-analysis showed positive correlations between PSR and behavior (Tukachinsky et al., 2020), although not in the area of health communication.

Based on these findings and given that reality TV characters can be perceived as similar to real-life friends, we propose that individuals who develop stronger PSRs with reality TV show characters will report more increase in exercise behavior over time than individuals who develop weaker PSRs (H2b).

## Parasocial Breakup and its Effects on Self-Efficacy and Behavior

Although research showed positive effects resulting from PSRs with media characters, problems might arise when the relationships end or dissolve. Those PSBUs describe a situation in which a media character with whom a viewer has formed a relationship goes off the air for whatever reason; for example, a character leaves the show, the show is stopped, or a viewer decides to stop watching (J. Cohen, 2003). Parasocial breakups, therefore, occur frequently in the everyday lives of viewers of E-E programs. As PSRs can positively impact health-relevant outcomes (F. Shen & Han, 2014), the ending of such relationships can have negative consequences. Particularly in the health sector, parasocial experiences with media personalities can be of special importance. If audience members orient themselves to media characters and build a relationship with them, this relationship is likely to give them hope and contribute to perseverance. If, however, these media characters drop out of the show, this breakup might lead to discontinuing the newly acquired behavior.

Empirical research on PSBU is limited so far. A systematic inventory of 60 years of research on parasocial phenomena showed that only 1.1% (*N* = 261) of empirical publications focused on PSBU (Liebers & Schramm, 2019). Of those, most studies addressed how PSBU affect audience members emotionally and whether this was similar to social breakups (J. Cohen, 2004; Eyal & Cohen, 2006; for exceptions, see E. L. Cohen & Hoffner, 2016; Lather & Moyer-Gusé, 2011). Furthermore, most studies focused on fictional PSBUs (J. Cohen, 2003, 2004) or did not investigate the effects of a sudden breakup or a breakup without warning. For the latter, participants were prepared because the show’s ending was previously discussed widely in the media (Eyal & Cohen, 2006). Findings showed that the experience of a PSBU in those cases is not as stressful as real social breakups. However, a PSBU can still be psychologically trying for the audience members and may lead to emotional distress (J. Cohen, 2003). This is especially true for audience members with high PSRs (Eyal & Cohen, 2006).

In sum, it has been shown that PSBUs have an effect on the emotional experience of the audience. Hence, it could be hypothesized that also other areas of their personal life become affected. Given that PSBUs and personal breakups show similar features, it seems legitimate to consult literature on social breakups in order to establish assumptions about the effects of PSBUs on determinants of exercise behavior. Research on romantic breakups, for example, found that individuals who experienced intense loss show increased depression (-like) symptoms (T. Field, 2011). Sadness, in turn, is associated with less motivation to be active, and healthy behaviors are replaced with unhealthy ones such as unhealthy food consumption (Garg & Lerner, 2013). If audience members lose their role model, as it goes off the air, they might be disappointed and even discouraged. In turn, pronounced PSBU distress might exhibit more self-regulatory failure in exercise practices compared to people with less PSBU distress. We can therefore assume that individuals experiencing PSBU distress more intensively report lower self-efficacy perceptions after the breakup than individuals experiencing less intense PSBU distress (H3a). Furthermore, we hypothesize that individuals experiencing more intense PSBU distress report less exercise behavior after the breakup than individuals experiencing less intense PSBU distress (H3b).

## Method

### Research design and procedure

Using the setting of the show TBL, we conducted a quasi-experimental longitudinal field study. PSRs are supposed to be stronger with media characters that are relevant to the audience members’ own situation, are similar to them (Q. Tian & Hoffner, 2010; Tukachinsky & Stever, 2018), and with whom they can identify (Eyal & Dailey, 2012). In addition, it might be that bonding with the portrayed characters is facilitated if overweight people relate easier to the topics the characters talk about than people with less weight (Sender & Sullivan, 2008). Therefore, we focused on overweight people in our sample. Participants were asked in a first questionnaire to indicate their height and weight. This information was used to calculate their BMI score by taking their weight in kilograms and dividing it by the square of their height in meters. One week after the first questionnaire, participants were asked to watch a first episode of TBL. Afterward, they filled out a second questionnaire. In the following 4 weeks, participants watched one episode of TBL per week and completed an online questionnaire before and after exposure. One week after the last episode, participants filled out a post-questionnaire where they were told about the study goal and the show’s final outcome. The episodes and questionnaires were available online, and the participants received reminders via email.

### Stimulus material

Participants watched five modified and shortened episodes of TBL (length approx. 30 minutes). In this health-related reality TV show, contestants compete to lose weight and win money (Domoff et al., 2012). For our study, we edited the episodes so that a female and a male character were put in focus to allow participants to form a PSR with either of them. In the first four episodes, they saw the characters struggling with the challenges in the show, supporting each other, and fighting against their overweight. In order to present the characters as ordinary people with whom the audience members can develop personal bonds, some private scenes showed the couple at home before participating in the show, conversations about relationship problems due to their overweight, or the couple reading emotional letters from their children. However, the main focus has been on the characters engaging in physical exercise. Since PSR is associated with the amount of exposure to the media character (Tukachinsky et al., 2020), both of the characters together cover 52% of the screen time in the first four episodes. At the end of the fourth episode, both characters had to leave the show because they lost less weight than their competitors (PSBU). The last episode depicted the show’s remaining participants without focus on a specific character. In doing so, we were able to keep the overarching narrative of TBL.

### Participants

Participants were recruited through a market research institute and received an equivalent of USD 14 for participating in all seven waves, which corresponds approximately with the average compensation for participation in university research projects. In order to be eligible for our study, participants had to be between 20 and 55 years old and living in Germany and have a BMI greater than 25. Within the first wave, the sample structure was representative of age and gender but only consisted of participants with a BMI larger than 25. At t_0_, more than 600 individuals finished the questionnaire. During the following 7 weeks, however, two-thirds decided to end their participation (t_0_: 603, t_1_: 357, t_2_: 298, t_3_: 263, t_4_: 248, t_5_: 218). This high dropout rate was expected as each wave was very time-consuming for the participants. A total of 198 participants were exposed to all five episodes and completed all seven questionnaires. After data cleansing, 149 participants remained in the sample (*M*_*BMI*_ = 30.42, *SD*_*BMI*_ = 6.04; 35.6% female, 63.8% male, 0.7% divers; *M*_*age*_ = 37.49; *SD*_*age*_ = 8.56, *n* = 148).

### Measures

Unless otherwise mentioned, all items presented below were measured using a five-point Likert-type scale from one (e.g., “not at all applicable to me” or “do not agree at all”) to five (e.g., “totally applicable to me” or “fully agree”). Descriptive statistics and internal consistencies are displayed in Table 1.

**Table 1.** Means, Standard Deviations, and Cronbach’s α of Study Variables.

	*N*	*M*	*SD*	Cronbach’s α
Parasocial relationship				
t_1_^a^	149	3.18	0.85	0.92
t_2_	148	3.16	0.80	0.92
t_3_	149	3.18	0.86	0.94
t_4_^b^	149	3.19	0.90	0.93
Parasocial breakup distress				
t_5_	148	2.00	0.95	0.94
Exercise behavior				
t_2_	148	41.52	31.93	—
t_3_	149	39.44	32.47	—
t_4_	146	39.92	32.28	—
t_6_	148	43.29	32.94	—
Perceived self-efficacy				Spearman-Brown coefficient^c^
t_1_	149	3.77	1.01	0.82
t_2_	147	3.93	0.89	0.79
t_3_	147	3.96	0.83	0.69
t_4_	149	4.00	0.84	0.81
t_6_	148	4.01	0.81	0.71

*Note.* t_1_ refers to the second wave of the study.

^a^ Item “I am thinking about [media character] even when I’m not watching an episode” not included.

^b^ Item “I am looking forward to seeing [media character] again in the next episode” not included.

^c^ Because perceived self-efficacy was measured with two items, the Spearman-Brown coefficient (split-half reliability) is reported instead of Cronbach’s α.

### Parasocial Relationship

After exposure to the first episode, participants were asked to indicate to which of the two characters mainly displayed in the episode they felt a closer connection. The selected media character represented their parasocial partner for the next few weeks, and her or his name was inserted in all questions related to character involvement. Data showed that 38.9% of the participants chose the female character, and 61.1% chose the male character. The majority of men (81.1%) chose the male character and, to a smaller extent, but still, the majority of the women (73.6%) chose the female character. The only person who identified as gender-diverse chose the female character.

Twelve items assessed parasocial relationship in questionnaires 2–5 (t_1_–t_4_) based on the scales developed by Hartmann et al. (2008) and by A. M. Rubin et al. (1985). Sample items include “I have the feeling that I know [the character] really well” or, “I think about [the character] even when I’m not watching the show.” Responses to these 12 items were averaged to create a composite measure of PSR, with higher values indicating higher PSRs.

### Parasocial Breakup Distress

Parasocial breakup distress was measured in questionnaires 6 and 7 (t_5_ and t_6_) using 13 items taken from J. Cohen, 2003; Eyal & Cohen, 2006. In line with the original scale, the items represent both an emotional (e.g., “Now that [the character] is off the air, I feel sad”) as well as a behavioral dimension (e.g., “Now that [the character] is off the air, I tend to think of [him/her] more often”). A reliability analysis was carried out on the PSBU distress scale comprising all 13 items. Cronbach’s alpha showed acceptable reliability, and most items appeared to be worthy for retention. However, two exceptions to this were items 8 (“Now that [the character] is off the air, I don’t miss [him/her] as much as I thought I would”) and 10 (“Now that [the character] is off the air, I found a different character to like”). Their deletion notably increased alpha. These items were negatively formulated, and probably participants overread the reversed phrasing. Hence, these two items were removed, and the remaining 11 items were averaged to form a composite measure, with higher values indicating more parasocial distress.

### Exercise Behavior

In questionnaires 3–7 (t_2_–t_6_), participants were asked to indicate how many times they performed three different kinds of exercise (i.e., strenuous, moderate, and mild/light) for more than 15 minutes during their free time within the last 7 days. A leisure activity score was built to assess our participants’ exercise behavior (Courneya et al., 2006; Godin et al., 1985, 1986; see Godin (2011), for the questions, classification of exercise intensity, and calculation of the weekly leisure activity score). Higher values indicate more intense physical activity. Values larger than 200 are almost impossible to reach during 1 week and were therefore identified as outliers and excluded from the analyses.

### Perceived self-efficacy

Self-efficacy was measured in all questionnaires (t_0_–t_6_) using two items assessing participants’ perceptions of self-efficacy with respect to measures and recommendations on exercise behavior and overweight (e.g., “I am able to exercise regularly; ” Witte, 1996). Responses to these items were averaged to create an index of self-efficacy, with higher values indicating stronger self-efficacy perceptions.

## Results

Repeated-measures analysis of variance (ANOVA) was used to examine the increase in PSR over time. Mauchly’s test indicated that the assumption of sphericity had been violated (*X*^2^ (5) = 48.14, *p* < .001), therefore degrees of freedom were corrected using Huynh–Feldt estimates of sphericity (ε = .81). Results showed that there was no increase in parasocial relationship over time, *F*(2.46, 361.95) = 0.13, *p* = 0.915, partial η^2^ = .001; *n* = 148 (H1 rejected).

As there was no significant change over time for PSR across all participants, dividing the sample into participants with high and low PSR would be reasonable. To validate this and, perhaps, to account for different types of development of PSR over time, we decided to build clusters of PSR (two-step cluster analysis as between-subjects variable) and use these clusters for the following analyses. For this, we considered the four measurement time points for PSR. A two-cluster solution resulted in being meaningful. The mean for the silhouette measure for cohesion and separation was at 0.6 (considered as “good”; Bacher et al., 2004). The first group (*N* = 70) indicated a high PSR already at the first measurement point and raised slowly over time (*M*_*T1*_ = 3.72; *M*_*T2*_ = 3.78; *M*_*T3*_ = 3.82; *M*_*T4*_ = 3.90). The second group (*N* = 78) showed a low PSR from the beginning which only slightly descended over time (*M*_*T1*_ = 2.69; *M*_*T2*_ = 2.61; *M*_*T3*_ = 2.60; *M*_*T4*_ = 2.57). Using those clusters for PSR (high with small ascent vs. low with small descent) for the following analyses and not a time-varying measure of the development of PSRs has important consequences for testing H2a and H2b. We hypothesized that participants who develop stronger PSRs would report a significant increase in self-efficacy perceptions (H2a) and in exercise behavior (H2b) than participants who develop weaker PSRs. However, with the clusters, these predictions cannot be tested, and the hypotheses need to be adjusted accordingly.

Thus, the modified H2a now predicts that participants with higher PSRs would report a significant increase in self-efficacy perceptions compared to the low PSR group. A mixed ANOVA was conducted with self-efficacy as the repeated measure and the clustered groups (low+descending vs. high+ascending PSR) as the between-subjects factor. Mauchly’s test indicated that the assumption of sphericity had been violated (*X*^2^ (5) = 51.41, *p* < .001). Therefore, degrees of freedom were corrected using Huynh–Feldt estimates of sphericity (ε = 0.79). The interaction between time and PSR was not significant, *F(*2.44, 348.33) = 0.30, *p* = .781, partial η^2^ = .002; *n* = 145, suggesting that developments in self-efficacy over time did not vary by PSR (H2a rejected).

Additionally, we estimated an unconditional linear growth curve model with the four measurements of self-efficacy to indicate how self-efficacy itself develops throughout exposure to TBL without taking any explanatory variables—but time—into account. The model fits the data well (see Table 2). The mean intercept and slope are statistically significant; the latter indicates that the mean trajectory of self-efficacy increased over time. The significant variance in the intercept reveals that some individuals have higher initial levels of self-efficacy while others have lower levels. However, the non-significant variance in the slope suggests the participants do not (significantly) differ with regard to the development of self-efficacy (rate of change) over time. There was also no significant covariance between slope and intercept, suggesting that the rate of self-efficacy change does not depend on their initial levels. Hence, the participants do show a small but significant increase in self-efficacy over the course of watching TBL.

**Table 2.** Development of Self-Efficacy Over Time. Unconditional Linear Growth Curve Model; Unstandardized Coefficients.

Model Results					
		Estimate	S.E.	Est./S.E.	Two-Tailed P-Value
S	With				
I		−0.013	0.021	−0.615	0.538
Means					
I		3.836	0.074	52.040	0.000
S		0.058	0.019	3.094	0.002
Variances					
I		0.592	0.097	6.124	0.000
S		0.008	0.009	0.908	0.364
Residual Variances					
Self-Efficacy t_0_		0.473	0.072	6.579	0.000
Self-Efficacy t_1_		0.178	0.030	5.970	0.000
Self-Efficacy t_3_		0.145	0.025	5.791	0.000
Self-Efficacy t_4_		0.115	0.033	3.538	0.000

*Note.* I = Intercept/Levels of Self-efficacy; S = Slope/Development of Self-efficacy (Index, see text) over time. AIC = 1147.651; BIC = 1174.687; Chi-square = 4.399, df = 5 p = .04934; CFI = 1.000; TLI = 1.000; RMSEA = 0.000; SRMR = 0.043.

H2b predicted that participants who developed stronger PSRs with the reality TV characters would show a greater increase in exercise behavior compared to those who developed weaker PSRs. As with H2a, this hypothesis cannot be tested exactly that way with the two clusters. Therefore, the modified H2b predicts that participants with higher PSR would show a greater increase in exercise behavior than the low PSR group. A mixed ANOVA was conducted with exercise behavior as the repeated measure and the clustered groups as the between-subjects factor. As the assumption of sphericity had been violated, *X*^2^ (2) = 14.05, *p* = .001, degrees of freedom were corrected using Huynh–Feldt estimates of sphericity (ε = 0.91). The interaction between time and PSR was not significant, *F*(1.86, 264.45) = 0.32, *p* = .712, partial η^2^ = .002, *n* = 144, suggesting that differences in exercise behavior over time did not vary by PSR (H2b rejected).

H3a predicted that individuals experiencing more intense PSBU distress would report lower self-efficacy perceptions after the breakup than individuals experiencing less intense PSBU distress. A linear regression analysis was performed with breakup distress as the independent variable and self-efficacy as the dependent variable. Because the assumption about the normal distribution of the residuals was not met, we used bootstrapping based on 1000 samples to compute robust estimates of *b*s and their confidence intervals (A. Field, 2013). In addition, we calculated robust standard errors (i.e., heteroskedasticity-consistent standard errors; HC4) because the assumption of homoscedasticity was violated (Hayes & Cai, 2007). Results showed that PSBU distress did not affect self-efficacy perceptions, *F*(1, 146) = 0.13, *p* = .715, *R*^*2*^ = .00; *B* = −0.03, *HC4* = 0.07; 95% CI [−.16, .10]; *n* = 148, (H3a rejected).

H3b predicted that individuals experiencing more intense PSBU distress would report lower exercise behavior after the breakup than those who experienced weaker PSBU distress. The assumption about the normal distribution of the residuals was not met, and we, therefore, used bootstrapping based on 1000 samples to compute robust estimates of *b*s and their confidence intervals. Results revealed no effect of PSBU distress on exercise behavior, *F*(1, 146) = 1.13, *p* = .289, *R*^*2*^ = .01; *B* = 3.04, *SE B* = 2.79; 95% CI [−2.34, 8.68]; *n* = 148 (H3b rejected).

## Discussion

This study set out to analyze the effects of PSRs with characters in a health-related reality TV show on health-relevant outcomes. Additionally, we were interested in how an audience member’s behavior would be affected when their favorite media character leaves the show. This study is among the rare longitudinal studies to measure the development and impact of PSRs with media characters over time (Liebers & Schramm, 2019; for an exception, see, Bond, 2020) and the first study to examine the effects of PSBUs on health-relevant behavior. Exploring these perspectives should help extend scholarship in PSR and breakup and bring forward the investigation of the E-E function of health communication.

Following theoretical assumptions as well as empirical findings, we expected PSRs with reality TV characters to increase over time and after repeated exposure. However, this hypothesis was not supported. There are several possible explanations for this result. First, due to the experimental setting, participants could not freely choose the TV show. Furthermore, they had to choose between two proposed characters as parasocial partners. Consequently, the participants were somewhat forced into the relationship with these specific characters, which might have hindered a natural development of a PSR. However, while participants were forced to choose a parasocial partner out of two media characters, they were not forced to actually develop a parasocial relationship with one of them. This means that participants could indicate in the questionnaire at any wave that they did not bond with the media character (PSR scale). As data shows, some participants indeed did not develop a PSR while others showed high levels of PSR from the beginning. This would indicate that it is possible for some people to enter into a PSR in a short time and without choosing completely freely the character. In addition, we tried to facilitate the development of PSRs as much as possible and important predictors discussed in the literature on PSRs were considered. For instance, participants watched the show and the characters repeatedly as the development of PSRs is based on repeated encounters (Horton & Wohl, 1956). The mere-exposure effect could have led to a more pronounced PSR. Additionally, the main characters had as much screen time as possible in order to allow the audience members to bond with them by vicariously engaging in the depicted activities. Indeed, a recent meta-analysis found that PSR was moderately associated with the amount of exposure to the media character (Tukachinsky et al., 2020). Furthermore, the participants were able to choose between a male and a female character because it has been found that men rather develop PSRs with same-sex characters and that women are generally more open to cross-sex PSRs (Eyal & Cohen, 2006). Lastly, perceived homophily is a strong predictor for PSR (Tukachinsky et al., 2020). Transferred to the context of the present study, we assumed that the relevance of the personal (health-related) situation is an important factor, and we, thus, only included overweight participants in our sample. This assumption is reinforced by Yoo (2013), who found that individuals who are more concerned with their weight watch more episodes of TBL. In our study, participants’ involvement with body weight was, on average, medium to high (*M* = 3.46, *SD* = 0.99; 1 = low involvement, 5 = high involvement). Furthermore, participants indicated they feel rather too heavy (*M* = 4.17, *SD* = .62; 1 = too thin, 5 = too heavy). Nevertheless, our results did not confirm the development of PSRs over time.

Second, we assumed TBL to be an appropriate TV show to allow the recipients to form PSRs because of the presumed authenticity of the media characters in reality TV shows. However, TBL has recently been criticized as being somewhat unrealistic, inaccurately portraying weight loss (B. J. Smith & Bonfiglioli, 2019) and because audience members realized the program’s manipulation (Holland et al., 2014). This might have impeded the development of PSRs.

Third, production features may also influence PSR development (Bond, 2020). For instance, research suggests that “breaking the fourth wall” and directly addressing the audience members can create the illusion of social interaction and increase PSR development (Tukachinsky & Stever, 2018). This is because directly addressing the audience increases PSI (Auter, 1992), and PSI and PSR are supposed to reinforce each other (Hu, 2016). In our study, the media characters never addressed the audience directly, which might have impeded the development of PSRs.

Fourth, the rather high dropout rate indicates that there may have been individuals who would perhaps have lower PSR at the beginning of the study, then may have developed an increasingly pronounced PSR over time and after repeated exposure. However, these individuals probably left the study early. Furthermore, since we based our analysis on individuals who participated in all waves, we might be missing those who had rather moderate PSR at the beginning of the show.

Based on the SCT, we argued that persuasive effects of E-E formats are largely driven by the audience members’ positive feelings toward and the involvement with the depicted media characters (i.e., PSR), as people learn from peers around them and adapt their behavior. Thus, such emotional bonds are supposed to increase attention to the behavior of the media characters, which, in turn, should facilitate observational learning (Moyer-Gusé, 2008; Tukachinsky & Tokunaga, 2013). We assumed that individuals who develop stronger PSRs would report a stronger increase in self-efficacy perceptions and in exercise behavior compared to individuals who develop weaker PSRs. As there was no increase in PSR over time and after repeated exposure, we decided to use clusters for the PSR variable (high+ascending vs. low+descending PSR) and not a dynamic measure of the development of PSRs. Consequently, the assumptions had to be rephrased. The modified hypotheses predicted that participants with higher PSRs would report more increase in self-efficacy perceptions and show a larger increase in exercise behavior compared to the low PSR group. Our results showed that PSRs with characters in the reality weight-loss competition had no influence on health-relevant outcomes such as exercise behavior and self-efficacy perceptions. This might indicate that PSRs are not as impactful as expected in this specific context. Although previous research showed that viewers generally appreciate the underlying concept of the show (i.e., weight loss through healthy eating and physical exercise; Thomas et al., 2007), a recent qualitative study using in-depth interviews found that that audience members perceived the portrayal of excessive exercise in the show to be unrealistic and that they were unsure whether the displayed physical activities would guide and inspire themselves (B. J. Smith & Bonfiglioli, 2019). It seems possible that our results are due to the fact that viewers were rather intimidated and demotivated by the activities displayed in the show. Thus, by showing contestants engaging in unrealistic exercise behavior, desired health outcomes were not promoted. Nevertheless, data showed a general increase of self-efficacy over time. This indicates that self-efficacy perceptions do increase over the course of watching TBL. Hence, there is some positive impact of watching the show. But this positive impact is not dependent on PSR but occurred due to other characteristics of the show or the mere exposure.

Although TBL markets itself as a show promoting a healthy lifestyle by encouraging audience members to lose weight, health professionals and researchers in the fields of public health and health communication raise concern about the message of the reality TV show. For instance, there is criticism that TBL promotes the stigma that being overweight or obese is an individual’s fault, that individuals should take personal responsibility for losing weight (Thomas et al., 2007), and that failure has to be taken as personally (Mocarski & Bissell, 2016). Furthermore, Silk and colleagues (2011) criticize that overweight and obese people are portrayed as immoral and irresponsible citizens and as a “problem” that needs to be solved. Research indeed found that after exposure to TBL, participants reported greater dislike of overweight and obese individuals (Domoff et al., 2012). Furthermore, some viewers felt that TBL gives the impression that people are not accepted in society if they do not lose weight. Some audience members even expressed discomfort while watching the show as contestants were exposed to public ridicule (Holland et al., 2014). Unfortunately, participants in the present study were not asked how they perceived the portrayals of overweight and obese people in TBL, and the question of whether the message that TBL transmits was perceived as rather discouraging than motivating cannot be answered with our data. However, after exposure to every episode, participants could indicate how they liked the episode in general. Data showed that the general liking of the episodes varied between so-so and good.

Another explanation why PSRs with characters of TBL had no influence on health-relevant outcomes is that some kind of reactance was triggered either due to our study design or due to exposure to TBL. Such defensive responses occur when individuals feel that their freedom is threatened, which might reduce the effectiveness of persuasive health-related messages (Brehm & Brehm, 1981; Dillard & Shen, 2005). However, models on E-E assume that audience members respond less with reactance to E-E because the persuasive intent is more concealed than in traditional persuasive messages (e.g., Moyer-Gusé, 2008; see, also, Ratcliff & Sun, 2020). If it was not reactance that was triggered, another negative effect might have contributed to our non-significant results. For instance, a meta-analysis on body satisfaction showed that the body image for women was significantly more negative after viewing thin media images than after viewing images of either average and plus-size models or objects such as cars and houses (Groesz et al., 2002). Transferred to TBL, this would indicate that when audience members are exposed to perceived unrealistic physical activity coupled with rapid weight loss, they compare themselves with a fanciful ideal, resulting in poor self-perception and body dissatisfaction or even feelings of guilt or shame. This can be problematic if there is an association between negative affect and overweight/obesity. Because the standard presumably established by TBL might make viewers feel bad about their weight and their exercise behavior, an undesired behavior could be promoted eventually.

A recently developed model proposes that people who self-identify as having overweight are more likely to experience psychological distress leading to behaviors that impair against health than those who do not (Robinson et al., 2020). In this model, positive or negative self-perception or body dissatisfaction are not playing a major role. However, the authors highlight that “the widespread and socially acceptable stigmatization of heavier body weight has negative consequences for people who identify as being overweight” (Robinson et al., 2020, p. 553) because those people would be aware that they have a characteristic that is stigmatized. This recognition may be associated with negative physical and mental health outcomes. The impact of self-identification of overweight on health outcomes is thus supposed to be mediated by internalization of stigma and social rejection concerns. Fear of social rejection and internalization of stigma, in turn, may increase psychological distress. As mentioned above, frequently experiencing psychological distress may negatively impact health-promoting behaviors (Robinson et al., 2020). This would indicate that merely being aware of having overweight or being reminded of it, for example, through a health-related reality TV show, can actually backfire and promote unhealthy behavior instead of physical exercise.

Regarding the effects of PSBU distress, we found that distress intensity had no impact on self-efficacy perceptions or exercise behavior. One possible explanation is that overall PSBU distress was relatively low in our sample (see Table 1), which indicates that the PSRs were not strong enough to be associated with the intensity of PSBU distress. However, additional correlation analyses showed a positive relationship between PSR and PSBU distress. Another explanation is that audience members knew that there are other candidates or that there will be another season with new candidates to form an attachment with (Schiappa et al., 2006), and this knowledge reduced the effects of PSBU distress.

## Limitations and Future Research

Despite the strength of a longitudinal research design, this study faced several limitations. First, the choice of TBL as a motivator of physical exercise can be criticized because the show has been described as “exploitative, embarrassing, and cruel” (Sender & Sullivan, 2008, p. 575). Furthermore, there has been criticism that the show is more interested in the entertainment value than in the educational intention (Mocarski & Bissell, 2016). For instance, practical tips on how to exercise at home are rare. The physical activities displayed in the show are extreme and cannot be carried out at home or in a gym. Hence, TBL might not provide audience members with a clear and concrete advice that they could perform exercises themselves.

Second, when assessing the parasocial partner, we asked participants to which of the two proposed characters they felt a closer connection. However, PSRs are more complex and can even be characterized by feelings of antipathy, disgust, or hatred (Hartmann et al., 2008). Thus, merely asking about a “closer connection” might not be an adequate indicator for a parasocial partner. Therefore, further research might assess a potential parasocial partner with a more sophisticated measure or explore the effects of naturally evolving PSRs without limiting the choice of the parasocial partner.

Although we found no increase in PSRs over time, our results showed that almost half of the participants showed strong PSRs from the beginning and maintained this relationship. It can be assumed that especially participants who could not relate to the characters at all did not continue with our study, and only those remained who had established some kind of parasocial relationship early. However, using the remaining individuals for the analysis makes the results all the more valid because the dropouts would not have watched further episodes of TBL, even in real-life situations. Nevertheless, results might differ when estimating models keeping not only the loyal or conscientious participants in the analysis who felt obliged to continue the study because of the incentives or because they felt to need to stand to their commitment.

Furthermore, it could be argued that exposure to TBL for 5 weeks might not be a sufficient time for audience members to develop a PSR, particularly if they did not choose such a relationship voluntarily. The literature on PSR does not specify how much time is needed for an audience member to develop a PSR. The amount of time probably depends on various factors, including personal and situational factors of the audience members, and there might be individual tendencies of viewers to develop PSRs with media characters (Tukachinsky & Stever, 2018). Regarding personal factors, research on narrative transportation suggests that the so-called transportability (i.e., the “chronic motivation and ability to become transported into a narrative, regardless of the content of the narrative”; Mazzocco et al., 2010, p. 362) plays an important role in becoming transported into stories and that individuals with higher transportability might be more persuaded (see, for example, Mazzocco et al., 2010). Thus, as there are individual differences in the likelihood of becoming transported into a narrative, there might as well be differences in the likelihood of developing PSRs with media characters due to favorable or unfavorable personality characteristics. For example, it has been shown that individuals who strive for intimacy show higher levels of PSRs than individuals with avoidant attachment styles (e.g., Cole & Leets, 1999; for a discussion on the role of various audience characteristics at different stages of the development of PSRs, see, Tukachinsky & Stever, 2018). Furthermore, if higher transportability would correlate with increased persuasion, a higher tendency to develop PSRs might also correlate with more increase in exercise behavior. Future studies could assess which factors increase the motivation and ability to develop PSRs in order to better understand the development of PSRs.

Additionally, one can imagine rather early decisions to bond with a character, and the PSR simply becomes more solid over time which might not be indicated by the classic PSR scales. For example, one can declare somebody as a true friend, but the reliability of the answer and the resistance to change might become more solid over time but not the evaluation of the friendship. Only a rare number of studies in communication research measure attitudes twofold—evaluation and potency or importance. Unfortunately, we were not able to apply both kinds of measurement due to restrictions of time and possible commitment issues. However, it could be assumed that, in particular, the potency or the importance of PSR develops over time—but not the mere evaluation, which is usually measured with the PSR scales. This perspective could be addressed in a future study. Nevertheless, our data showed that, for individuals with generally higher levels of PSRs, the intensity of PSR increases slightly over time. For individuals with generally lower levels of PSRs, PSR intensity slightly decreases.

Lastly, applying a screening process to participation in the study with the Transtheoretical Model (TTM; Prochaska & Velicer, 1997) could help future studies with both the high dropout rate and the lack of effects on self-efficacy perceptions and exercise behavior. The TTM is a dynamic and situational health behavior theory that stresses the fact that behavioral change takes place in several stages. Individuals may find themselves in different phases; therefore, a health-related message is most effective if tailored to the situational stage at which an individual is. Thus, individuals should already want a behavior change before participating in such a study. The stages of change model also suggest that self-efficacy is an important predictor of people’s motivation to engage in health-related behaviors (DiClemente & Prochaska, 1998). Results of our study showed that self-efficacy perceptions increased over time. This would indicate that participants did not lack the confidence that they can successfully perform physical exercises. Theoretically, these individuals possess the ideal basis for adopting a healthy lifestyle in the near future.

In spite of its limitations, the present study is among the rare experimental longitudinal studies on the development and impact of PSRs with media characters (Liebers & Schramm, 2019). Therefore, a study similar to this one should be carried out to address the above-mentioned limitations.
